# Visceral adipose tissue volume effect in Crohn's disease using reduced exposure CT enterography

**DOI:** 10.1002/acm2.14235

**Published:** 2023-12-07

**Authors:** Sara A. Hunter, Mark E. Baker, Justin M. Ream, David E. Sweet, Nicholas A. Austin, Erick M. Remer, Andrew Primak, Jennifer Bullen, Nancy Obuchowski, Wadih Karim, Brian R. Herts

**Affiliations:** ^1^ Imaging Institute – Cleveland Clinic Cleveland Ohio USA; ^2^ Siemens Medical Solutions USA Malvern Pennsylvania USA; ^3^ Department of Quantitative Health Sciences – Cleveland Clinic Cleveland Ohio USA

**Keywords:** Crohn disease, CT scan, inflammatory bower disease, radiation dosage, visceral adipose tissue, x‐ray

## Abstract

**Purpose:**

The purpose of this investigation was to assess the effect of visceral adipose tissue volume (VA) on reader efficacy in diagnosing and characterizing small bowel Crohn's disease using lower exposure CT enterography (CTE). Secondarily, we investigated the effect of lower exposure and VA on reader diagnostic confidence.

**Methods:**

Prospective paired investigation of 256 CTE, 129 with Crohn's disease, were reconstructed at 100% and simulated 50% and 30% exposure. The senior author provided the disease classification for the 129 patients with Crohn's disease. Patient VA was measured, and exams were evaluated by six readers for presence or absence of Crohn's disease and phenotype using a 0–10‐point scale. Logistic regression models assessed the effect of VA on sensitivity and specificity.

**Results:**

The effect of VA on sensitivity was significantly reduced at 30% exposure (odds radio [OR]: 1.00) compared to 100% exposure (OR: 1.12) (*p* = 0.048). There was no statistically significant difference among the exposures with respect to the effect of visceral fat on specificity (*p* = 0.159). The study readers’ probability of agreement with the senior author on disease classification was 60%, 56%, and 53% at 100%, 50%, and 30% exposure, respectively (*p* = 0.004). When detecting low severity Crohn's disease, readers’ mean sensitivity was 83%, 75%, and 74% at 100%, 50%, and 30% exposure, respectively (*p* = 0.002). In low severity disease, sensitivity also tended to increase as visceral fat increased (ORs per 1000 cm^3^ increase in visceral fat: 1.32, 1.31, and 1.18, *p* = 0.010, 0.016, and 0.100, at 100%, 50%, and 30% exposure).

**Conclusions:**

While the interaction is complex, VA plays a role in detecting and characterizing small bowel Crohn's disease when exposure is altered, particularly in low severity disease.

## 1 INTRODUCTION

Lower exposure CT has been the subject of numerous investigations over the last 10−15 years, sparked by the concern that excessive and repeated examinations may lead to the development of more cancers.^1^ Patients with chronic conditions, such as Crohn's disease, often required repeated examinations.^2^, ^3^, ^4^, ^5^ Thus, many institutions have reduced CT enterography (CTE) exposure in an effort to reduce patient lifetime exposure. Prior investigations have concluded that lower exposure CTE may not substantially affect efficacy^6^, ^7^, ^8^; however, these investigations have only focused on Crohn's disease detection and did not study disease characterization. They have also not investigated the effect of lower radiation exposure exams on the reader confidence in both detecting and characterizing the disease that might lead to reporting equivocation.

Visceral adipose tissue volume (VA) has also been the subject of much investigation over the last several years, most often focusing on detecting and quantifying its volume and its significance in disease process and outcome.^9^ One omission in investigating visceral adipose volume is the important role it plays as an inherent contrast agent in the abdomen. All radiologists have experienced the difficulty of evaluating the bowel in the absence of VA.^10^ The more VA present between bowel loops, the easier it is to identify bowel wall abnormalities. Over the last several years at our institution, utilizing lower exposure CTE, we have noticed that it is more difficult to identify and correctly characterize the imaging based morphologic phenotype of small bowel Crohn's disease when the VA is low.^11^ We have speculated that lower VA reduces our ability to both diagnose and characterize Crohn's disease and often lead to more equivocal reports.

The purpose of this investigation was to investigate effect of VA on reader efficacy in diagnosing and characterizing small bowel Crohn's disease using the Society of Abdominal Radiology (SAR)/American Gastroenterological Association/Society of Pediatric Radiology morphologic phenotypes at lower exposure CTE.^11^ Secondarily, we investigated the effect of lower exposure and VA on reader diagnostic confidence.

## 2 METHODS

### 2.1 Patient selection and population

This was a prospective, paired, HIPAA compliant, IRB approved (informed consent waived) investigation of clinically ordered CTE.

Assuming 5−6 readers, 3−4 exposure levels, using a 0−10‐point scale for the presence or absence of disease, an initial power analysis was performed in order to assess the number of patients necessary to show a significant difference. This showed that 118 patients with small bowel Crohn's disease would be needed to obtain 80% power to detect a correlation of 0.3 or larger. Seven readers were subsequently recruited and six completed the assessment.

Consecutively over a 27‐month period, from October 2016 through January 2019, and when a research technologist was available (generally 2−3 days per week), the raw data from CTE's performed at our main CT facility was collected. The prolonged patient accrual process was due to patients meeting reference standard inclusion criteria.

Data from 345 patients were initially obtained, with 89 patients subsequently excluded who did not have adequate confirmation of Crohn's or absence of Crohn's disease. The reference standard for disease status was ileocolonoscopy, pathological and surgical confirmation, or compelling clinical evidence, as indicated by long history of Crohn's disease confirmed by gastroenterology or colorectal surgery. In the final study population of 256 patients, there were 129 patients with confirmed Crohn's disease and 127 patients without Crohn's disease. Of the confirmed cases of Crohn's disease, 94 had ileocolonoscopic confirmation, 23 had pathologic/surgical confirmation, and 12 had compelling clinical evidence for Crohn's disease. All patients without Crohn's disease had a negative ileocolonoscopy. Further, all these normal patients had no other small bowel diseases that might be confused with Crohn's disease. In the 256 patients included in the study, there were 135 women and 121 men with a median age of 44 (interquartile range [IQR] 32, 59) and a median body mass index (BMI) of 25 (IQR 22, 30). Of the 129 patients with Crohn's disease, there were 62 women and 67 men, with a median age of 43 (IQR 32, 59) and a median BMI of 25 (IQR 21, 29) (*p* = 0.95, 0.17, and 0.47, respectively, for age, gender, and BMI) (Table 1). Of the 127 patients without Crohn's disease, there were 73 women and 54 men with a median age of 45 (IQR 32, 58) and a median BMI of 25 (IQR 22, 30). The similarities in patient baseline characteristics between the with Crohn's and without Crohn's groups were incidental. The indications for the CTE in these patients with Crohn's disease were often multiple and included abdominal pain/distention, diarrhea/constipation, inflammatory bowel disease, colitis, and enteritis. The indications for the CTE in patients without Crohn's disease were abdominal pain/distention (45 patients), inflammatory bowel disease (IBD)/colitis (42 patients), diarrhea/constipation (16 patients), and other (2 patients).

**TABLE 1:** Summary of patient characteristics, overall and stratified by disease status.

	Entire sample	Without Crohn's	With Crohn's	*p*‐value^a^
# Patients	256	127	129	
Age	44 [32, 59]	45 [32, 58]	43 [32, 59]	0.95
Male^†^	121 (47.3)	54 (42.5)	67 (51.9)	0.17
BMI	25 [22, 30]	25 [22, 30]	25 [21, 29]	0.47
Visceral adipose tissue volume (cm^3^)	1973 [842, 3852]	1885 [677, 3462]	2145 [1003, 3995]	0.13
kVp	100 [100, 110]	110 [100, 110]	100 [100, 110]	<0.001^*^
QRM^b^				0.30
100	209 (81.6)	100 (78.7)	109 (84.5)	
150	47 (18.4)	27 (21.3)	20 (15.5)	
CTDIvol	5 [4, 7]	6 [4, 8]	4 [3, 6]	<0.001^*^

Unless otherwise specified, data are medians ± interquartile range (IQR). CTDIvol, CT dose index‐volume; QRM, quality reference mAs.

^a^ Comparison of patients with and without Crohn's.

^b^ Data are numbers of patients, with percentages in parentheses.

* Indicates statistical significance.

### 2.2 CT enterography technique, reconstruction, and lower exposure simulation

All CTEs were performed using neutral enteric contrast media. Starting 1 h before the examination, and after an IV was placed, the patients ingested 1000 mL (two 500 mL bottles) of Breeza (Beekley Medical, Bristol, CT). Approximately 10 min before scanning, the patients ingested 250 mL of water. A total of 238 (93%) of the 256 patients took the full volume of oral contrast prior to CTE, with 92% compliance among patients with Crohn's and 94% of the patients without Crohn's disease. All patients took at least half volume of contrast. All the exams were reviewed by the senior author and were deemed diagnostic.

All patients were scanned on a 192‐slice, dual‐source CT scanner (SOMATOM Force, Siemens Healthineers, Forchheim, Germany) using version VA50A software. Automated kVp selection (CARE kV, Siemens Healthineers) was activated with 120 Reference kVp; slider bar setting 7; and variable Quality Reference mAs (qmAs) depending on patient's weight: (1) 100 Ref mAs for patients < 200 lbs; (2) 150 Ref mAs for 200 lbs < patients < 300 lbs; and (3) 200 Ref mAs for 300 lbs < patients < 350 lbs. Other acquisition parameters were as follows: 192 × 0.6 mm detector collimation, 0.5 s gantry rotation time and 0.6 pitch. Full‐exposure images were reconstructed at 3 mm slice thickness using Bf40 kernel with iterative reconstruction (ADMIRE, strength 3) using an offline research workstation (ReconCT with SimNoise plugin, Siemens Healthineers, Forchheim, Germany). ADMIRE strength 3 was chosen for the full exposure data as this is what is used clinically at our institution. In addition, noise was inserted into the raw data from the full‐exposure scans to reconstruct images simulating 50% and 30% exposure scans. This algorithm is similar to that which has been described and previously validated^12^ that inserts noise into the sinogram space, accounting for properties such as bowtie filter, automated exposure control, and electronic noise. The simulated 50% and 30% exposure is based on mean scanner output in terms of median CT dose index‐volume (CTDIvol). 50% exposure was used given the results of prior investigations^8^ and 30% exposure was added given other investigations.^13^ The noise simulator did not include an additional layer of iterative reconstruction for the 50% or 30% simulated exposures as this would add an extra layer of complexity to the study.

All examinations were performed after the intravenous administration of iodinated contrast media (iohexal [Ominipaque 300], GE Healthcare, Wauwatosa, WI), 2 mL/kg (150 mL maximum dose) at an injection rate of 2.5 mL/s. At full inspiration, patients were scanned 90 s after the start of contrast injection (portal venous phase occurs at this point due to very fast scanning parameters).

In the 256 patients the median kVp was 100 (IQR 100, 110) and the median CTDIvol was 5 (IQR 4, 7). In the patients with Crohn's disease the median kVp was 100 (IQR 100, 110) and in patients without Crohn's disease the median kVp was 110 (100, 110) (*p* < 0.001). In the patients with Crohn's disease the median CTDIvol was 4 (IQR 3, 6) and in patients without Crohn's disease the median CTDIvol was 6 (IQR 4, 8) (*p* < 0.001) (Table 1).

### 2.3 Visceral adipose tissue volume assessment

The VA was measured using Aquarius Intuition software (v.4.4.13), TeraRecon (Durham NC). The volume was automatically measured for each axial slice of the CTE, starting at the level of the mid‐liver (portal vein at porta hepatis) to the level of the mid pelvis at the level of the acetabular roof. Total VA was the sum of volume from all these slices.

In the entire sample, the median VA was 1973 cm^3^ (IQR 842, 3852). In the patients without Crohn's disease the median VA was 1885 (IQR 677, 3462). In the patients with Crohn's disease the median VA was 2145 (IQR 1003, 3995) (*p* = 0.13) (Table 1).

### 2.4 Reader experience and training

The six readers had the following levels of experience: three abdominal subspecialist staff radiologists with 28, 27, and 6 years of experience; one abdominal radiologist in fellowship training; two fourth year radiologists in training with interest in abdominal imaging. The recruitment strategy included finding readers of varied experience levels, who had interest in abdominal radiology, and availability to read the necessary 768 CTE.

Prior to participating in the study, the senior author [MEB], an international expert on Crohns disease and CTE, provided each reader tailored feedback on evaluating CTE and differentiating Crohn disease phenotypes. Subsequently, readers were given a detailed set of instructions with illustrations before image interpretation that included how to identify active Crohn's disease, the presence of stricturing and penetrating disease and examples of the imaging based, morphologic phenotypes based on Bruining et al.^11^ Additionally, after reading and reviewing the instructions with the senior investigator, each reader was given nine test cases, not included in the study, in order to evaluate their understanding of the imaging based, morphologic phenotypes of Crohn's disease (see below). Readers were encouraged to review no more than 30 cases in one sitting and reviewed the set of randomized cases over a period of 3−9 months, depending on individual availability outside routine work commitments.

### 2.5 Image analysis

The 127 normal small bowel CTEs and 129 Crohn's disease CTEs were reconstructed at 100% and simulated 50% and 30% exposure. The resulting list of 768 CTE examinations were randomized and individualized for each reader. Both the axial and coronal images were available and were viewed on a TeraRecon Aquarius (Durham, NC) platform, with the ability to window and level the images. Readers first determined if the images were normal or showed Crohn's disease using a 0−10‐point confidence scale, with 0 representing definite normal small bowel and 10 representing definite Crohn's disease. A rating of 6 or above was considered positive for Crohn's disease. If 5 or below, the exam was considered normal. If the rating was 6 or greater, the reader was asked to determine the imaging based morphologic phenotype. The reader could choose one or more possible phenotypes, using a confidence scale of 0−10, with 0 representing definite absent and 10 representing definite present per phenotype. The total confidence levels for all phenotypes considered had to add to 10. Finally, readers had to rate the subjective quality of the exam from 0 to 10, with 0 representing a non‐diagnostic exam and 10 representing optimal quality. 0−10 point scale was used as a compromise between using a 0−5 point scale and the quasi‐continuous 100 point scale.^14^

From these scales, three pieces of information was being collected from the readers. First, the binary decision of presence of absence of disease, used to give an estimate of sensitivity and specificity; second, the amount of confidence in their decision, used to calculate the ROC; and finally, the further classification of phenotype if the reader thought Crohn's disease was present.

### 2.6 Disease presence and phenotype

All examinations were reviewed by the senior author to confirm the presence and absence of Crohn's disease and provide the disease classification for the patients with Crohn's disease. Furthermore, all the normal CTEs were reviewed to confirm that there were no small bowel abnormalities which could be misconstrued as small bowel Crohn's disease. The senior author is a member of the SAR Crohn's Disease Focus Panel, and was the main originator of the imaging based, morphologic phenotypes. When present, the disease was characterized using the imaging based, morphologic phenotypes described by SAR/American Gastroenterological Association/Society of Pediatric Radiology consensus paper^11^: active inflammation without luminal narrowing; active inflammation with luminal narrowing; stricture with active inflammation; stricture with active inflammation & penetrating disease; stricture without active inflammation; Aand stricture without active inflammation & penetrating disease. For the subset analysis, low severity disease was considered active inflammation without or with luminal narrowing.^11^ If the patient had a prior resection for Crohn's disease and had no findings of Crohn's disease on the current imaging and with ileocolonoscopy, they were considered normal. Thus, the Crohn's disease without imaging signs of active inflammation phenotype was not an option for the reader. Among the 129 patients with Crohn's disease, the imaging based morphologic phenotypes were as follows: five had active disease without luminal narrowing, 45 active disease with luminal narrowing, 47 stricture with active inflammation, and 32 stricture with active inflammation and penetrating disease.

### 2.7 Statistical methods

For all analyses involving sensitivity and specificity, a reader confidence score of 6 or higher was considered positive. Logistic regression models were used to assess the effect of VA on reader sensitivity and specificity in detecting Crohn's disease. Odds ratios (ORs) were estimated via logistic regression; specifically, they were the exponentiated beta coefficient for the relevant predictor. When results were pooled across the readers, generalized estimating equations (GEE) were employed to account for the clustered/dependent nature of the data. GEE is a method to estimate model parameters when there is potential for unmeasured correlation between observations (in this study due to multiple readers providing results on the same patient). An exchangeable correlation structure was used, as we assumed there was a single between‐reader correlation common across potential pairs of readers. An interaction term (VA * exposure level) was used to test the hypothesis that the effect of VA depends on the exposure level. A significance level of 0.05 was applied to all hypothesis tests.

In order to explore the relationship between VA and the readers’ mean area under the receiver operating characteristic (ROC) curve, VA was categorized. The categories were selected after looking at the models of sensitivity and specificity. The readers’ mean area under the ROC curve (AUC) was then estimated for each combination of VA category and exposure. One of the statisticians selected the boundaries for VA after looking at the results from the models of sensitivity and specificity and at histograms of VA, blinded to the resulting AUCs. All analyses were performed in R version 4.2.2.^15^ GEE were computed using package “geepack”.^16^

## 3 RESULTS

### 3.1 Effect of exposure on sensitivity, specificity, and AUC

In general, the relationship between exposure level and reader sensitivity was statistically significant (*p* = 0.017) (Table 2). The two pairwise comparisons that were significant were 100% versus 50% exposure and 100% versus 30% exposure, with 95% CIs for the difference in sensitivity being [0.001, 0.069] and [0.017, 0.084], respectively. In general, the relationship between exposure level and reader specificity was not statistically significant (*p* = 0.404). In general, the relationship between exposure level and the AUC was not statistically significant (*p* = 0.311).

**TABLE 2:** Readers’ mean sensitivity, specificity, and area under the received operating characteristic (ROC) curve.

	100% Exposure	50% Exposure	30% Exposure	*p*‐value
Sensitivity	89%	86%	84%	0.017^a^
Specificity	87%	88%	90%	0.404
AUC	0.934	0.918	0.922	0.311

*N* = 127 patients without Crohn's and 129 patients with Crohn's. AUC, area under the curve.

^a^ Indicates statistical significance.

### 3.2 Effect of visceral adipose tissue volume on AUC

After looking at models estimating the effect VA on sensitivity and specificity, subjects were binned into three categories based on their VA: <1000 cm^3^ (*n* = 78), 1000−5000 cm^3^ (*n* = 148), and >5000 cm^3^ (*n* = 30). The AUC was highest at 100% exposure with a VA > 5000 cm (0.949) and lowest at 30% exposure with a VA > 5000 cm (0.897) (Table 3). At VA of <1000 cm, the AUC for 100% exposure was 0.946, for 50% was 0.929, and for 30% was 0.947.

**TABLE 3:** Readers’ mean area under the received operating characteristic (ROC) curve at each combination of exposure and visceral adipose volume.

		Visceral adipose volume (cm^3^)		
		<1000	1000–5000	>5000
Exposure	100%	0.946	0.924	0.949
	50%	0.929	0.912	0.900
	30%	0.947	0.914	0.897

*N* = 78, 148, and 30 patients with visceral adipose <1000 cm^3^, 1000−5000 cm^3^, and >5000 cm^3^, respectively.

### 3.3 Effect of visceral adipose tissue volume and exposure on sensitivity

**FIGURE 1:**
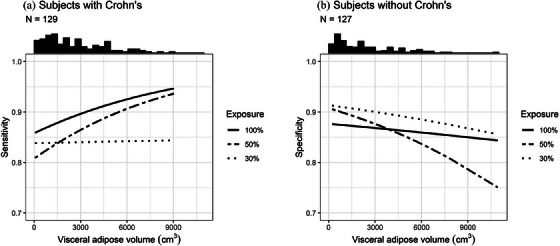
Predicted sensitivity (a) and predicted specificity (b) as a function of visceral adipose tissue volume, stratified by exposure level. Results pooled across 6 readers. Histogram of visceral adipose volume in each sample displayed at top of plot.

Within each exposure group there was no statistically significant association between VA and reader sensitivity (Table 4, Figure 1a). However, the effect of VA on sensitivity was significantly reduced at 30% exposure (OR: 1.00) compared to 100% exposure (OR: 1.12) (*p* = 0.048). There was no statistically significant difference between 50% and 100% exposure with respect to the effect of VA on sensitivity (*p* = 0.165).

**TABLE 4:** Association between visceral adipose volume and odds of true positive among patients with Crohn's, stratified by exposure.

	100% Exposure		50% Exposure		30% Exposure	
	OR^a^	*p*‐value	OR^a^	*p*‐value	OR^a^	*p*‐value
Reader 1	1.23	0.18	1.42	0.03	1.16	0.24
Reader 2	1.83	0.19	1.38	0.23	1.13	0.46
Reader 3	1.03	0.91	1.37	0.17	0.99	0.93
Reader 4	1.07	0.49	1.06	0.57	0.85	0.10
Reader 5	1.09	0.57	1.12	0.41	1.12	0.47
Reader 6	1.11	0.53	1.02	0.90	0.96	0.70
Pooled	1.12	0.17	1.14	0.13	1.00	0.995

*N* = 129 subjects. OR, odds ratio.

^a^ Odds ratio for a 1000 cm^3^ increase in visceral adipose volume.

### 3.4 Effect of visceral adipose tissue volume and exposure on specificity

No statistically significant association between VA and reader specificity was observed at any of the exposures (Table 5, Figure 1b). There was no statistically significant difference among the exposures with respect to the effect of VA on specificity (*p* = 0.159).

**TABLE 5:** Association between visceral adipose volume and odds of true negative among patients without Crohn's disease, stratified by exposure (*N* = 127 subjects).

	100% Exposure		50% Exposure		30% Exposure	
	OR^a^	*p*‐value	OR^a^	*p*‐value	OR^a^	*p*‐value
Reader 1	1.13	0.44	0.96	0.75	0.89	0.41
Reader 2	1.04	0.73	1.01	0.93	0.89	0.34
Reader 3	0.84	0.09	0.98	0.87	0.98	0.85
Reader 4	0.85	0.28	0.68	0.006	0.93	0.66
Reader 5	1.14	0.40	0.82	0.04	1.06	0.69
Reader 6	0.93	0.60	1.06	0.74	0.91	0.55
Pooled	0.97	0.64	0.90	0.10	0.94	0.51

OR, odds ratio.

^a^ Odds ratio for a 1000 cm^3^ increase in visceral adipose volume.

### 3.5 Effect of visceral adipose tissue volume and exposure on study reader agreement with senior author's classification of disease

**FIGURE 2:**
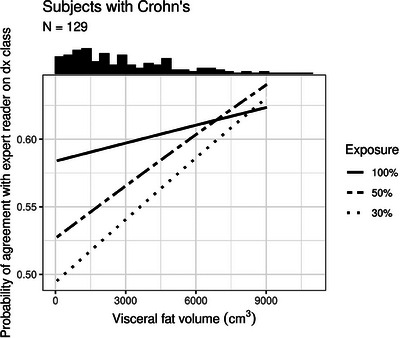
Predicted probability of study reader agreement with the expert reader on disease classification as a function of visceral fat volume, stratified by exposure level. Results pooled across six study readers. Histogram of visceral fat volume displayed at top of plot.

The senior author provided the disease classification for the 129 patients with Crohn's disease. A study reader was considered to agree with the senior author on disease classification if he or she gave a confidence score greater than 5 (0–5 score) to the disease category designated by the senior reader. The study readers’ probability of agreement with the expert reader on disease classification was 60%, 56%, and 53% at 100%, 50%, and 30% exposure, respectively (*p* = 0.004). The probability of agreement with the senior reader on disease classification tended to increase as VA increased, but this effect was not statistically significant (ORs per 1000 cm^3^ increase in VA: 1.02, 1.05, and 1.06, *p* = 0.717, 0.330, and 0.290, at 100%, 50%, and 30% exposure). (Table 6, Figure 2). There was no statistically significant interaction between exposure and VA with respect to their effect on probability of study reader agreement with the senior reader on disease classification (*p* = 0.490). There was good inter‐reader agreement among the six study readers with respect to the presence/absence of Crohn's disease (Fleiss’ Kappa = 0.71, 0.70, and 0.69 for the 30%, 50%, and 100% exposure levels, respectively).

**TABLE 6:** Association between visceral fat volume and odds of study reader agreement with the expert reader on disease classification among patients with Crohn's*, stratified by exposure.

	100% Exposure		50% Exposure		30% Exposure	
	OR^a^	*p*‐value	OR^a^	*p*‐value	OR^a^	*p*‐value
Reader 1	1.11	0.255	1.08	0.398	1.09	0.315
Reader 2	1.09	0.416	1.11	0.303	1.15	0.171
Reader 3	0.96	0.647	0.96	0.615	1.08	0.414
Reader 4	0.99	0.901	1.01	0.917	0.85	0.092
Reader 5	1.07	0.446	1.28	0.014	1.25	0.020
Reader 6	0.92	0.379	0.96	0.626	1.03	0.738
Pooled	1.02	0.717	1.05	0.330	1.06	0.290

*N* = 129 subjects. OR, odds radio.

^a^ Odds ratio for a 1000 cm^3^ increase in visceral fat volume.

### 3.6 Effect of visceral adipose and exposure on sensitivity in patients with low severity Crohn's disease

**FIGURE 3:**
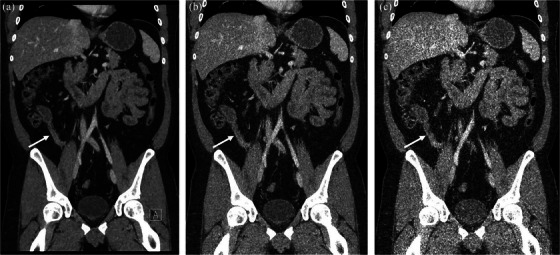
Coronal images of low severity Crohn's disease with 100% (a), and simulated 50% (b), and 30% (c) doses, shown at identical window and level settings and iterative reconstruction settings (ADMIRE, strength 3). Active inflammatory Crohn's disease with luminal narrowing of the terminal ileum (arrows) were confirmed by ileocolonoscopy and biopsy. This examination was given multiple low confidence ratings by interpreting readers.

**FIGURE 4:**
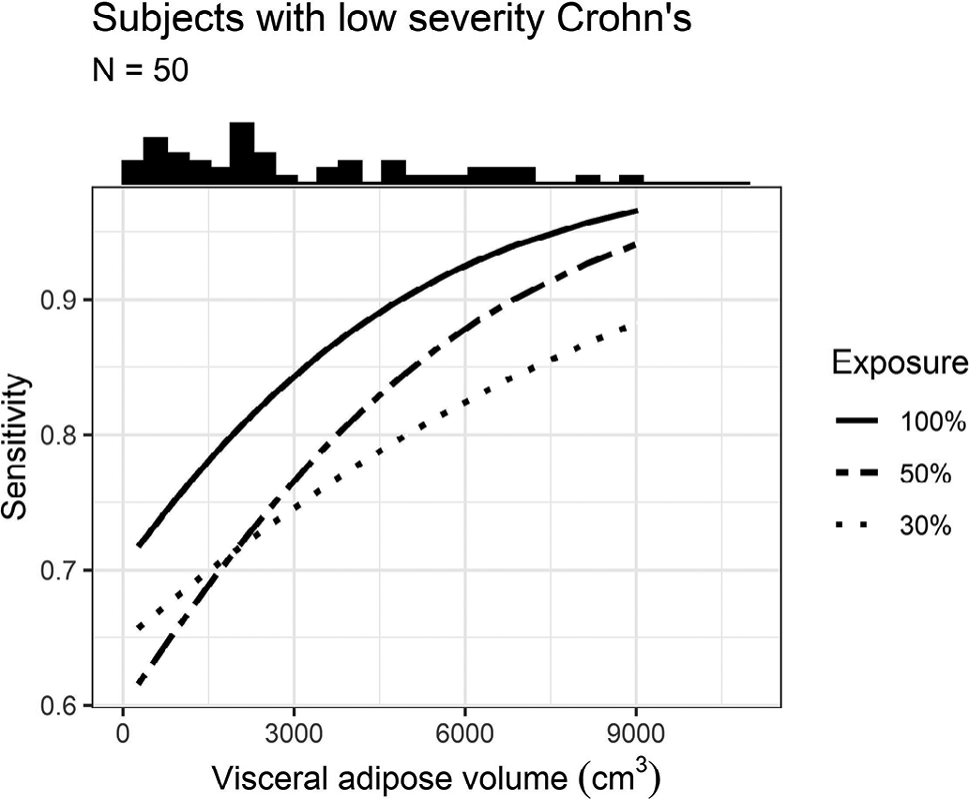
Predicted sensitivity as a function of visceral fat volume, stratified by exposure level, among patients with subtle level of disease (i.e., severity score of 1 or 2). Results pooled across six readers. Histogram of visceral fat volume displayed at top of plot.

This analysis focuses on the subgroup of 50 patients with subtle disease (i.e., active disease without and with luminal narrowing). Readers’ mean sensitivity was 83%, 75%, and 74% at 100%, 50%, and 30% exposure, respectively (*p* = 0.002) (Figure 3). Sensitivity increased as VA increased (ORs per 1000 cm^3^ increase in visceral fat: 1.32, 1.31, and 1.18, *p* = 0.010, 0.016, and 0.100, at 100%, 50%, and 30% exposure) (Table 7, Figure 4). There was no statistically significant interaction between exposure and VA with respect to their effect on sensitivity (*p* = 0.130).

**TABLE 7:** Association between visceral fat volume and odds of true positive among patients with low severity Crohn's, stratified by exposure.

	100% Exposure		50% Exposure		30% Exposure	
	OR^a^	*p*	OR^a^	*p*	OR^a^	*p*
Reader 1	1.37	0.095	1.76	0.010	1.25	0.168
Reader 2	1.99	0.161	1.95	0.121	1.33	0.117
Reader 3	1.61	0.218	1.91	0.070	1.22	0.252
Reader 4	1.38	0.048	1.15	0.296	1.02	0.851
Reader 5	1.17	0.508	1.28	0.196	1.80	0.104
Reader 6	1.19	0.330	1.14	0.353	1.08	0.571
Pooled	1.32	0.010	1.31	0.016	1.18	0.100

*N* = 50 subjects. OR, odds radio.

^a^ Odds ratio for a 1000 cm^3^ increase in visceral fat volume.

### 3.7 Effect of visceral fat and exposure on subjective image quality

**FIGURE 5:**
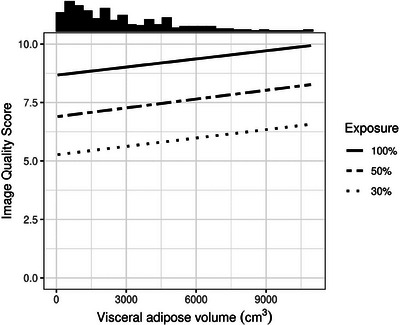
Predicted image quality score as a function of visceral fat volume, stratified by exposure level. Results pooled across six readers. Histogram of visceral fat volume displayed at top of plot.

The median (IQR) image quality score was 9 (8–10), 8 (6–8), and 6 (4–7) at 100%, 50%, and 30% exposure, respectively (*p* < 0.001). Subjective image quality increased as VA increased (mean increase per 1000 cm^3^ increase in VA: 0.12, 0.13, and 0.12, *p* < 0.001 at 100%, 50%, and 30% exposure) (Figure 5). There was no statistically significant interaction between exposure and VA with respect to their effect on subjective image quality (*p* = 0.910).

### 3.8 Effect of visceral adipose tissue volume and exposure on low reader confidence

**FIGURE 6:**
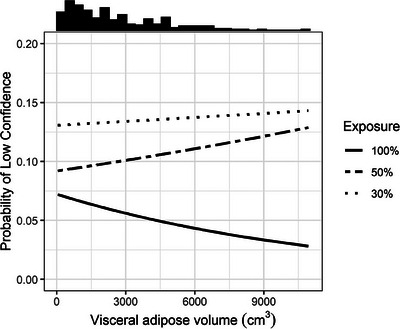
Predicted probability of low reader confidence (i.e., score of 4, 5, or 6) as a function of visceral adipose tissue volume, stratified by exposure level. Results pooled across six readers. Histogram of visceral adipose volume in each sample displayed at top of plot.

On average, readers gave a score of low confidence (4, 5, or 6) in 6%, 10%, and 13% of cases at 100%, 50%, and 30% exposure, respectively (*p* < 0.001). There was no statistically significant association between VA and low reader confidence at any of the exposures (*p* = 0.240, 0.380, and 0.740 for 100%, 50%, and 30% exposure, respectively) (Figures 1, 6). There was no statistically significant interaction between exposure and VA with respect to their effect on low reader confidence (*p* = 0.190). Interestingly, at both 100% and 50% exposure, the probability of a low confidence score increased slightly with increasing VA. Conversely, at 30% exposure, the probability of a low confidence score decreased with increasing VA.

## 4 DISCUSSION

Our investigation shows that exposure and VA play a complex role in the efficacy of detecting and morphologically characterizing Crohn's disease. At 100% and 50% exposure, sensitivity increased as VA increased, but not statistically. At 30% exposure, the effect of VA was much less. Our interpretation of this is that the effect of noise on sensitivity at 30% exposure outweighs the effect of VA. The dose modulation curves on our CT scanners do not increase linearly as patient soft tissue attenuation increases but flattens out at larger patient circumference. Thus, at higher visceral adipose volumes, noise increases given lower tube outputs. Further, the analysis of the correct classification of the morphologic phenotype of the disease shows that as exposure goes down, readers are less accurate in correctly characterizing the disease process. There was a tendency to increase the likelihood of correctly characterizing the disease as visceral adipose volume increased, but not statistically. Nonetheless, VA likely plays some role at lower exposure levels because the effect of VA on sensitivity was significantly reduced at 30% exposure when compared to 100% exposure.

Interestingly, the AUC was lowest at 50% dose for patients with VA volumes < 1000 cm^3^; conversely, there was no significant difference in the AUC between the 1000 and 5000 cm^3^ or >5000 cm^3^ VA volume groups for either 50% or 30% exposure. In fact, what this study likely shows is that the AUC is dependent on both the VA tissue and image noise, each affecting sensitivity and specificity to different degrees. It was our previous observation that we had difficulty identifying Crohn's disease in low VA volume patients which led to this study. When comparing sensitivity and specificity, the unanticipated finding described presently is likely due to a falsely higher sense of confidence at the 50% compared to 30% exposure; thereby increasing the number of incorrect diagnoses for low VA volume patients.

To our knowledge, no other investigation of low exposure CTE has investigated the effect of either the exposure level or the amount of VA in the morphologic characterization of Crohn's disease. Our data show that as exposure decreases, the ability of readers to characterize the disease significantly decreases from 100% exposure to 30% exposure. The data also show a tendency for lower visceral adipose volumes to affect the characterization. For patients with low severity Crohn's disease sensitivity increased with increasing VA at all exposure levels. Further, at all exposures, sensitivity was lower for cases with low severity Crohn's disease compared to the entire cohort. Less severe, more subtle disease appears to be more difficult to identify with lower exposure and in patients with lower VA (Figure 4). These are important findings as more subtle disease detection, especially when present out of the reach of an endoscope, is vital in the care of these patients.

Sensitivity decreased with the decreasing exposure, as expected. As exposure decreased, readers significantly chose lower confidence scores, with 1 out of 5 studies in the 30% dose group (Figure 6). While we did not assess the actual radiologic report in these cases, the confidence scores reflect the level of equivocation in diagnosis. Our findings suggest that lower exposure studies may lead to report equivocation. Equivocation in reporting is common and hard to measure but is a real phenomenon.^17^ When faced with an examination with excessive noise, the radiologists introduce ambiguity into their report and have higher rates of pseudolesions and missed pertinent findings.^7^, ^8^, ^18^ This often leads to confusion or misinterpretation by the clinician and, possibly, unnecessary additional work‐up or treatment.^19^

It is unfortunate that our findings did not show a statistically significantly relationship between VA, exposure and disease detection and characterization. This may be because there were unequally apportioned examinations in low, medium and high VA cohorts, containing 78, 148, and 30 patients, respectively (Table 3). Our results may also have been affected by only six readers, in terms of both limited power and potential bias. We faced a formidable challenge in recruiting even six radiologists to review nearly 800 CTEs. Nonetheless, the readers had a variety of experience levels, allowing for expected interreader variability. Another potential limitation of the study was the implementation of the same level of iterative reconstruction for the full and simulated reduced dose exposures instead of increasing the ADMIRE for lower simulated exposures. However, we chose not to alter the ADMIRE level as higher levels of iterative reconstruction create a “plastic‐like” image texture, which is not desirable for clinical use and would add additional complexity to the study.^20^

Given the trend that higher VA improves Crohn's disease detection and characterization, knowledge of the VA would be helpful in prescribing the appropriate scanning parameters. Dual energy (DE)/spectral topograms are now commercially available on Philips spectral (dual‐layer) detector scanners (IQon and Spectral CT 7500) (Phillips Healthcare, Andover, Massachusetts) and on the Siemens Alpha photon‐counting detector CT (Siemens Healthineers, Erlangen, Germany) (requires a research license for now). This approach is equivalent to dual x‐ray absorptiometry (DEXA) as both techniques use two x‐ray acquisitions at different energy/spectra. The DE/spectral topo technique has been shown to provide similar results to DEXA for bone mineral density (BMD) analysis.^21^, ^22^ It has also been shown that DEXA is capable of measuring VA.^23^, ^24^ Therefore, as the DE/spectral topo approach is equivalent to DEXA and DEXA can measure VA, then the DE/spectral topo technique could measure VA as well. If these capabilities were operationalized, radiologists could more cogently protocol patients based upon their estimated VA. An alternative to this would be to estimate the VA using a single slice through the abdomen. Several investigations have shown that one single slice can reasonably estimate the VA in patients, though there are varying opinions on which anatomic site for VA estimation is the most accurate in both the literature^25^, ^26^ and among our internal physicians and physicists.

In conclusion, we have shown that while the interaction is complex, the VA likely plays a role in detecting and characterizing Crohn's disease when exposure is altered. Sensitivity decreases with decreasing exposure and VA. Further, sensitivity for detecting less severe disease decreases with decreasing exposure. Lastly, confidence in distinguishing Crohn's disease presence from normal decreases with decreasing exposure. Knowledge of VA should inform x‐ray tube exposure parameters in CTE examinations in patients with suspected Crohn's disease.

## AUTHOR CONTRIBUTIONS

**Sara A. Hunter**: Formal analysis; investigation; writing—original draft; writing—review and editing. **Mark E. Baker**: Conceptualization; methodology; formal analysis; investigation; writing—original draft; writing—review and editing; funding acquisition; supervision. **Justin M Ream**: Methodology; formal analysis; investigation; writing—review and editing. **David E. Sweet**: Formal analysis; investigation; writing—review and editing. **Nicholas A. Austin**: Formal analysis; investigation; writing—review and editing. **Erick M. Remer**: Conceptualization; methodology; formal analysis; investigation; writing—review and editing. **Andrew Primak**: Methodology; writing—review and editing. **Jennifer Bullen**: Methodology; formal analysis; investigation; writing—review and editing; resources. **Nancy Obuchowski**: Formal analysis; investigation; writing—review and editing; resources. **Wadih Karim**: Writing—review and editing; resources. **Brian R. Herts**: Conceptualization; methodology; formal analysis; investigation; writing—review and editing; funding acquisition; supervision.

## CONFLICT OF INTEREST STATEMENT

The authors declare no conflicts of interest.
